# Serum soluble urokinase-type plasminogen activator receptor as a biological marker of bacterial infection in adults: a systematic review and meta-analysis

**DOI:** 10.1038/srep39481

**Published:** 2016-12-19

**Authors:** Wentao Ni, Yuliang Han, Jin Zhao, Junchang Cui, Kai Wang, Rui Wang, Youning Liu

**Affiliations:** 1 Department of Respiratory Diseases, Chinese PLA General Hospital, Beijing 100853, China; 2 Department of Neurology, Chinese PLA 305 Hospital, Beijing 100017, China; 3 Department of Clinical Pharmacology, Chinese PLA General Hospital, Beijing, 100853, China

## Abstract

The serum concentration of soluble urokinase-type plasminogen activator receptor (suPAR) reflects immune activation. We performed a meta-analysis to evaluate the usefulness of suPAR for the diagnosis and prognosis of bacterial infections. PubMed, Embase and Cochrane Library databases were searched for studies reporting the detection of suPAR in adult patients with bacterial infections. Seventeen studies were selected from 671 studies. The pooled sensitivity and specificity of suPAR for diagnosing infection were 0.73 and 0.79, respectively, and the area under the summary receiver operating characteristic curve (AUC) was 0.82. Subgroup analyses revealed suPAR showed similar AUC values for diagnosing sepsis and bacteremia, but the AUC for differentiating sepsis from systemic inflammatory response syndrome (SIRS) was only 0.68. Elevated suPAR levels were significantly associated with a high risk of death, with a pooled risk ratio of 3.37 (95% confidence interval, 2.60–4.38). The pooled sensitivity and specificity for predicting mortality were 0.70 and 0.72, respectivfely, with an AUC of 0.77. Serum suPAR could be a biomarker for the diagnosis and prognosis of bacterial infection, but it is relatively ineffective for differentiating sepsis from SIRS. Further investigation is required to evaluate whether using of suPAR in combination with other biomarkers can improve diagnostic efficacy.

The urokinase-type plasminogen activator (uPA) system is composed of a proteinase, uPA receptor (uPAR) and an inhibitor. The uPA system is involved in pericellular proteolysis, cell migration and tissue remodeling^1^. Soluble uPAR (suPAR) is the soluble form of uPAR, and is a glycoprotein with a molecular weight of 55–60 kDa. Under normal physiological conditions, uPA and uPAR are predominantly expressed by neutrophils, monocytes, macrophages and activated T-cells, and the serum concentration of suPAR is relatively stable throughout the day^2^,^3^. However, when inflammatory cells are activated by cytokines, the expression of uPAR can be up-regulated, thus elevating the serum levels of suPAR^4^. It has been demonstrated that the serum concentrations of suPAR can be increased during inflammatory and infectious diseases, such as arthritis, liver fibrosis, human immunodeficiency virus (HIV) infection, bacterial infection and malaria, reflecting the activation of the immune system^4^,^5^,^6^,^7^,^8^.

Therefore, suPAR might be a useful biological marker for diagnosing patients with systemic inflammation and infection. Several studies have described its potential for the prediction of infection^9^. Additionally, a high suPAR level has been shown to be associated with a poor prognosis of infections^1^. The review of Backes *et al*., which included a limited number of studies, evaluated the usefulness of the suPAR levels for diagnosing and prognosing systemic inflammation or infection^10^. Their review reported that suPAR showed potential as a promising prognostic marker for critically ill patients, but had little diagnostic value for sepsis^10^. However, the precise diagnostic and prognostic value of suPAR in bacterial infection remains unclear.

Recently, considerably more well-designed and well-executed studies with larger sample sizes investigating the diagnostic and prognostic value of suPAR in infections have been published. To establish the overall diagnostic and prognostic value of suPAR for adult patients with bacterial infections, we searched currently available publications and performed a meta-analysis.

## Methods

### Search strategy and selection criteria

We systematically searched studies indexed until June 10, 2016, in the PubMed, Embase and Cochrane Library databases. The search terms used were: (“soluble urokinase receptor” OR “urokinase plasminogen activator receptor” OR “suPAR” OR “soluble uPAR” OR “soluble uPA receptor”) AND (“infection” OR “systemic inflammation” OR “SIRS” OR “bacteremia” OR “sepsis” OR “septicemia” OR “septicaemia” OR “septic shock”). Additionally, the reference lists of the selected articles were reviewed manually to obtain potentially relevant articles. No language restrictions were applied.

Studies were considered eligible if they reported the diagnostic and/or prognostic usefulness of suPAR in adult patients suffering from suspected or defined bacterial infections. The bacterial infections were either confirmed by a microbiological test or diagnosed from their clinical manifestation. The diagnosis of sepsis and systemic inflammatory response syndrome (SIRS) met the criteria of the American College of Chest Physicians/Society of Critical Care Medicine^11^. Because we focused on evaluating the usefulness of serum suPAR as a biological marker of bacterial infection in adults, studies enrolling patients <18 years old, and studies reporting infections caused by pathogens other than bacteria, such as HIV, malaria, tuberculosis and fungi were excluded. A 2 × 2 contingency table was constructed based on the results of the selected studies. Reviews, conference abstracts, expert opinions, editorials, animal experiments and studies without enough data were also excluded. Two authors (NW and HY) independently performed the search and selection process. Any disagreement was discussed and resolved by the third author (CJ).

### Ethical approval

Ethical approval was not required in this study.

### Data extraction and quality assessment

Data from each included study were independently extracted by 2 authors (NW and HY). The following baseline information was extracted: authors and country where the study was performed, year of publication, study design, sample size, baseline characteristics of the study population (age, sex, underlying conditions, and intensive care unit [ICU] admission), mortality, and the time point of measurement of suPAR levels. With regards to outcomes of interest, the optimal cut-off threshold, values for sensitivity, specificity, true-positive, true-negative, false-positive, and false-negative, and the area under the receiver operating characteristic (ROC) curve (AUC) were extracted from each study.

This meta-analysis was conducted in accordance with the guidelines of the Preferred Reporting Items for Systematic Reviews and Meta-Analyses (PRISMA) statement. The quality and bias of the included studies were assessed using the Quality Assessment of Diagnostic Accuracy Studies (QUADAS) checklist by 2 independent authors (NW and HY). This quality assessment tool included 14 questions (each of which is scored as yes, no, or unclear) with a maximum score of 14 12.

### Statistical analysis

STATA (version 12.0) (StataCorp, College Station, TX) and Meta-Disc software were used for the statistical analyses of data. *P* values < 0.05 were considered statistically significant. Publication bias was tested by Deek’s funnel plot. Threshold effects on the diagnostic accuracy of suPAR were evaluated by the Spearman correlation coefficient between the logit of sensitivity and the logit of 1 – specificity. If no threshold effect existed, the bivariate random-effects regression model was used to calculate the pooled sensitivity, specificity, diagnostic odds ratio (DOR), positive likelihood ratio (PLR), and negative likelihood ratio (NLR). We also constructed the summary receiver operating characteristic (SROC) curve by plotting individual and summary points of sensitivity and specificity to assess overall diagnostic accuracy. In addition, relative risk (RR) was calculated to assess the usefulness of suPAR for predicting mortality, which was pooled by either a fixed-effect or random-effect model based on the DerSimonian and Laird method.

The *Q* test and *I*^*2*^ index were calculated to assess between-study heterogeneity. *I*^*2*^ > 50% was considered to be a substantial degree of heterogeneity among studies. Except for assessing the threshold effect, other sources of potential heterogeneity were explored by subgroup and univariate meta-regression analyses.

## Results

**Figure 1:**
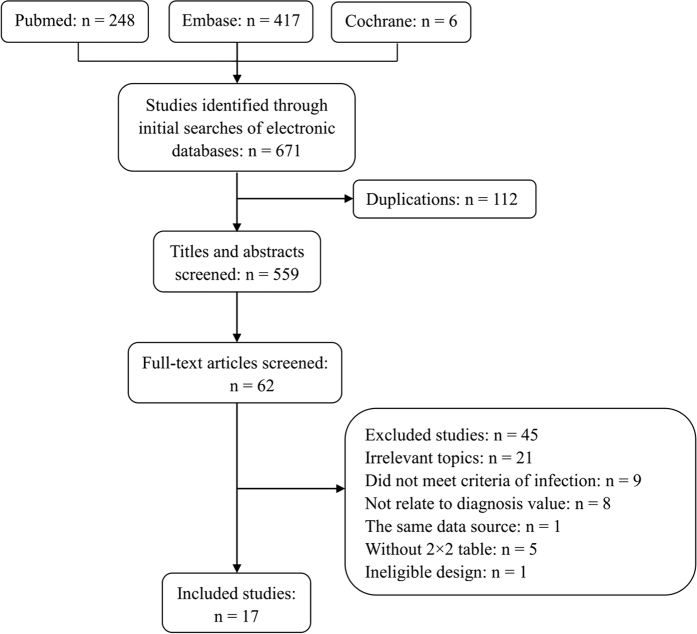
Flow chart of study selection.

The primary search identified 671 studies derived from the 3 databases. Of those 671 studies, 112 were eliminated because of repetition, and 559 were disregarded based on evaluation of their title or abstract, thereby leaving 62 studies to be scrutinized by a full-text review. No additional relevant articles in the bibliographies of the selected articles were identified. Finally, 17 studies were included in this systematic review. The detailed search and study selection process is shown in Fig. 1.

### Characteristics of the included studies

Among the 17 eligible studies, 9 studies including 1237 patients reported the usefulness of suPAR for diagnosing infections^13^,^14^,^15^,^16^,^17^,^18^,^19^,^20^,^21^, and 10 studies including 3041 patients reported its usefulness for predicting mortality^14^,^21^,^22^,^23^,^24^,^25^,^26^,^27^,^28^,^29^. The characteristics of the studies are presented in Table S1. One study had a retrospective design^18^, and the others had a prospective design. On average, the overall QUADAS scores of the included studies met 11 of the 14 criteria, suggesting that these studies were of a high quality. Of the 9 studies reporting the diagnostic value of suPAR, 3 recruited patients with bacteremia and control groups with negative blood culture tests^16^,^18^,^19^, and 7 recruited patients with sepsis and well-matched control groups with similar profiles of age and sex^13^,^14^,^15^,^16^,^19^,^20^,^21^. Additionally, 4 studies investigated the diagnostic value of suPAR in differentiating sepsis from SIRS^16^,^19^,^20^,^21^. Of the 10 studies reporting the value of suPAR in predicting mortality, 7 recruited patients suffering from sepsis^14^,^21^,^25^,^26^,^27^,^28^,^29^, and 3 recruited patients with bacteremia^22^,^23^,^24^. The serum level of suPAR was measured using an enzyme-linked immunosorbent assay kit (ViroGates, Birkerød, Denmark) in most of the included studies. The optimal cut-off threshold of each study was retrospectively determined based on the ROC curve. The mean cut-off for suPAR in the included studies where it was used for the diagnosis of infections was 6.4 ng/ml (range: 2.7–9.5 ng/ml), and the mean cut-off where it was used for mortality prediction was 10.0 ng/ml (range: 6–12.9 ng/ml).

### Diagnostic value of suPAR for infections

**Figure 2:**
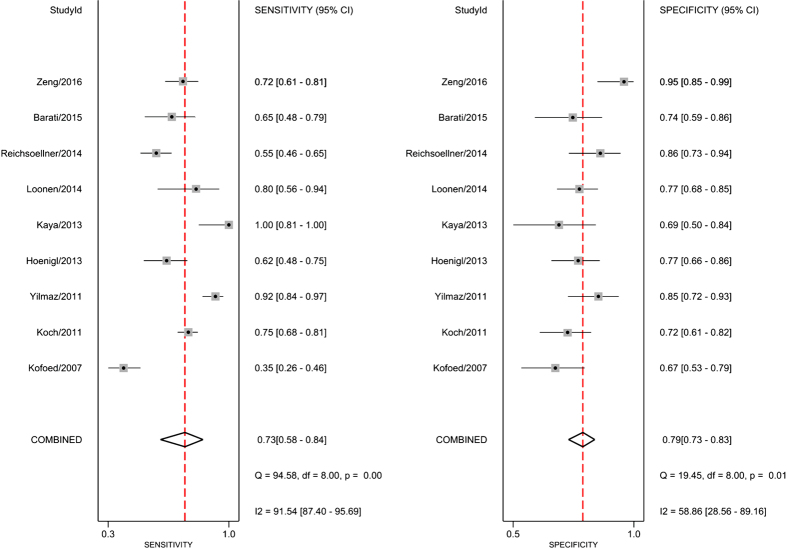
Forrest plot of the sensitivity and specificity of suPAR for the diagnosis of bacterial infections.

**Figure 3:**
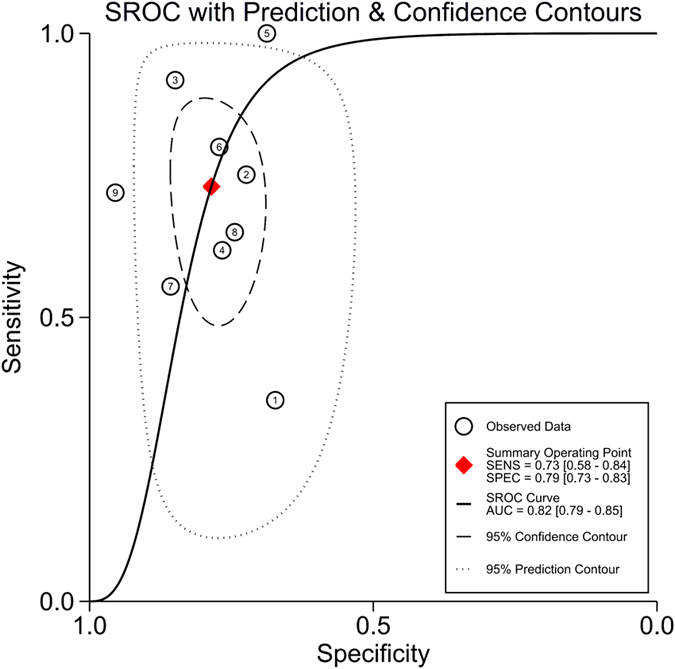
Summary receiver operating characteristics curve for studies evaluating the value of suPAR for the diagnosis of bacterial infections. SEN, sensitivity; SPE, specificity.

As shown in Figure S1, no significant publication bias was detected by Deek’s funnel plot (*P* = 0.39). Additionally, no significant difference in the threshold effect was observed (Spearman correlation coefficient = −0.03; *P* = 0.93). By using the bivariate random-effects regression model, the pooled sensitivity and specificity of suPAR in diagnosing infections were 0.73 [95% confidence interval (CI), 0.58–0.84; *I*^*2*^ = 91.54%, *Q* = 94.58 (*P* < 0.01)] and 0.79 [95% CI, 0.73–0.83; *I*^*2*^ = 58.86%, *Q* = 19.45 (*P* = 0.01)], respectively (Fig. 2). The PLR and NLR were 3.4 (95% CI, 2.4–4.7) and 0.34 (95% CI, 0.21–0.57), respectively. The DOR was 10 (95% CI, 5–22), and the overall AUC was 0.82 (95% CI, 0.79–0.85; Fig. 3), indicating a moderate diagnostic accuracy.

Substantial heterogeneity exists among the included studies. Thus, we used univariate meta-regression and subgroup analyses to explore the sources of heterogeneity in sensitivity and specificity. The clinical setting, sample size, type of infection, prevalence, tested sample and QUADAS score were used as covariates. Meta-regression analysis revealed that the clinical setting and QUADAS score significantly accounted for the heterogeneity of sensitivity, and no tested variables accounted for the heterogeneity of specificity (Figure S2). A subgroup analysis is presented in Table 1. suPAR showed a similar usefulness for diagnosing sepsis and bacteremia, with an area under the SROC curve of 0.82. However, its diagnostic performance in differentiating sepsis from SIRS was not optimal, with an AUC of 0.68 (95% CI, 0.64–0.72).

### Usefulness of suPAR for predicting mortality in cases of infection

**Figure 4:**
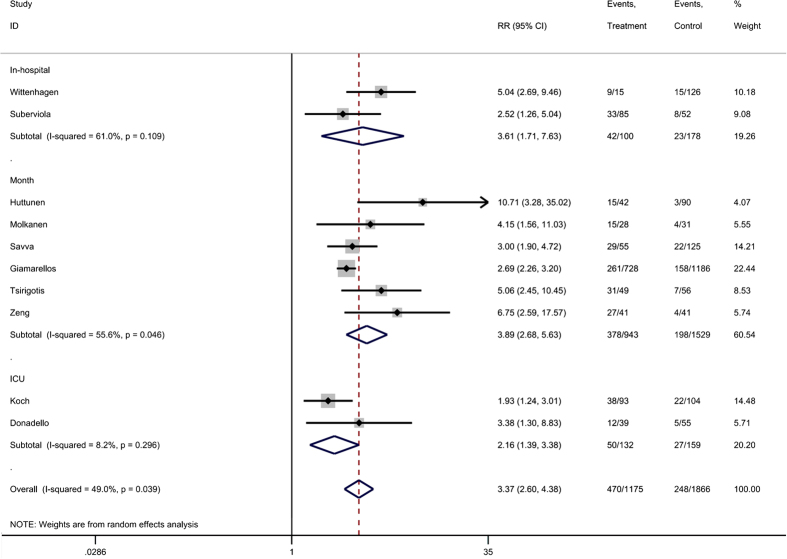
Forest plot of suPAR to predict mortality in bacterial infections. RR, risk ratio.

The pooled results of 10 included studies showed that an elevated suPAR level was associated with a higher risk of death due to infection (Fig. 4). The RR of each study ranged from 1.93 to 10.71, and the pooled RR were 3.61 for in-hospital mortality, 3.89 for 1-month mortality and 2.16 for ICU mortality. Owing to the moderate heterogeneity between studies, a random-effect model was used to pool RR estimates.

**Figure 5:**
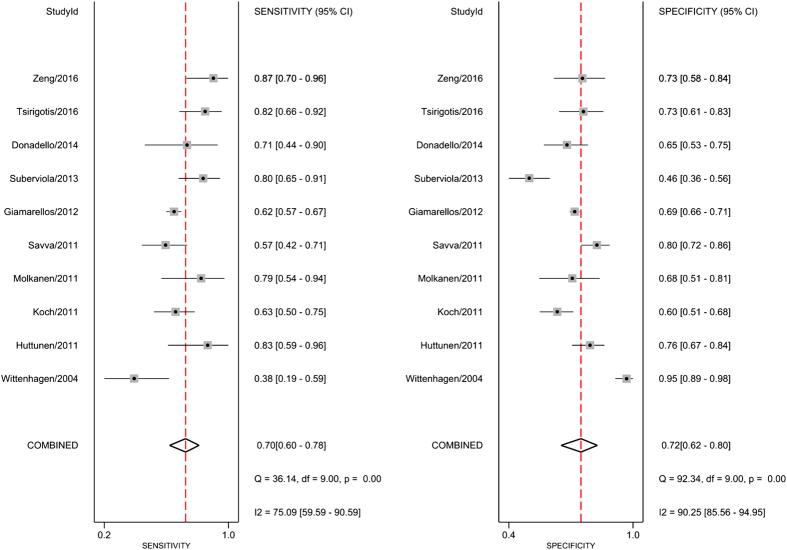
Forrest plot of the sensitivity and specificity of suPAR for the prediction of mortality in bacterial infections.

**Figure 6:**
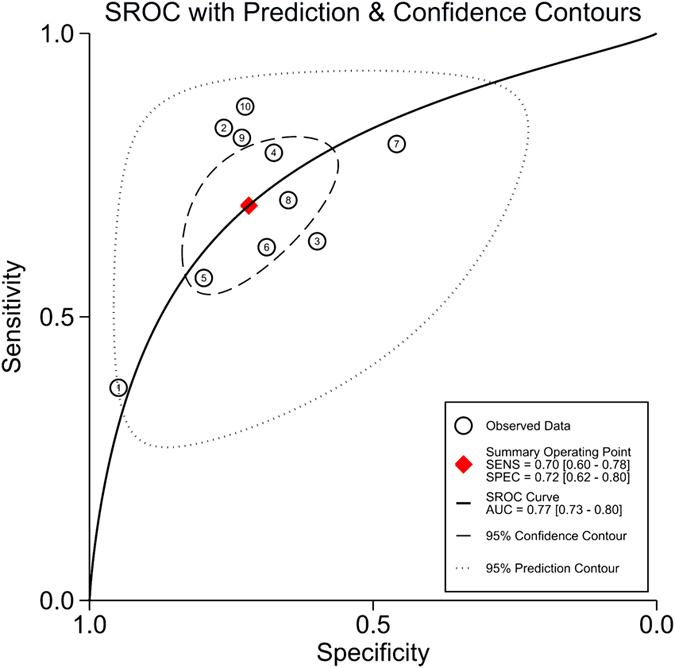
Summary receiver operating characteristics curve for studies evaluating the value of suPAR for the prediction of mortality in bacterial infections. SEN, sensitivity; SPE, specificity.

The Spearman correlation coefficient of these studies was 0.21 (*P* = 0.56), indicating that no significant threshold effect existed. However, Deek’s funnel plot showed there was a publication bias (*P* = 0.03; Figure S3). As displayed in Fig. 5, the estimated sensitivity and specificity of suPAR for predicting mortality of infection were 0.70 [95% CI, 0.60–0.78; *I*^*2*^ = 75.09%, *Q* = 36.14 (*P* < 0.01)] and 0.72 [95% CI, 0.62–0.80; *I*^*2*^ = 90.25%, *Q* = 92.34 (*P* < 0.01)], respectively. The DOR was 6 (95% CI, 4–9), and the AUC was 0.77 (95% CI, 0.73–0.80; Fig. 6), suggesting that the efficiency of suPAR was moderate for the prediction of mortality among patients with infection.

The study design, clinical setting, sample size, type of infection, prevalence, tested sample, mortality and QUADAS score were used as factors in the meta-regression analysis to assess the source of heterogeneity among studies. The findings revealed that: study design significantly contributed to the heterogeneities of both sensitivity and specificity (Figure S4). The results of the subgroup analysis are shown in Table 1.

## Discussion

Bacterial infections, such as sepsis and bacteremia, are the major causes of mortality and morbidity in critically ill patients^30^. Accurate and timely diagnosis to initiate proper anti-infective therapy and early screening of high-risk populations is mandatory to improve the clinical course and outcome. Culturing samples has been considered as the ideal standard, but it is time-consuming and lacks sensitivity and specificity^31^. Biomarkers can help to detect the presence of a bacterial infection, and they are useful for monitoring the evolution of the infectious process^27^. Therefore, clinicians are continuously seeking biological markers to differentiate infectious from non-infectious conditions. In this meta-analysis, the pooled data of 9 studies including 1237 patients showed that the overall AUC of suPAR for the diagnosis of bacterial infection was 0.82. The subgroup analysis found that the AUCs for diagnosing bacteremia and sepsis were also both 0.82. These results suggest that serum suPAR level might be a useful diagnostic criterion for bacterial infections. Additionally, the pooled results showed that an elevated suPAR levels was associated with a higher risk of death, with an AUC of 0.77, indicating a moderate prognostic usefulness of suPAR for bacterial infections.

Sepsis is a heterogeneous syndrome commonly caused by the response of the immune system to invasive bacterial infections^32^. Currently, serum C-reactive protein (CRP) and procalcitonin (PCT) have been evaluated routinely for sepsis diagnosis. The level of CRP can increase significantly during acute inflammation, but its specificity for sepsis diagnosis is relatively low^9^. In earlier studies, PCT was found to be a reliable marker for bacterial infections, with more sensitivity and specificity than CRP^33^. However, the meta-analysis performed by Tang *et al*.^34^ showed that the diagnostic performance of PCT for differentiating sepsis from other non-infectious causes of SIRS was not optimal. The mean values of sensitivity and specificity were both 71% (95% CI, 67–76), and the AUC was 0.78 (95% CI, 0.73–0.83)^34^. In our study, although suPAR had a moderate value for discriminating sepsis and bacteremia from non-infectious conditions, the subgroup analysis showed that the accuracy of suPAR for differentiating sepsis from SIRS in critically ill adult patients was low (AUC = 0.68). suPAR failed to exhibit a higher sensitivity than the other biomarkers used for discriminating sepsis from SIRS, such as PCT, soluble triggering receptor expressed on myeloid cells-1 (sTREM-1), and presepsin^34^,^35^,^36^.

By far, no biomarkers with ideal accuracy have been identified for monitoring the course and predicting the outcome of infections or sepsis. Peschanski *et al*. found that PCT levels at admission were moderately accurate in identifying the outcome in septic patients, with 51% sensitivity and an AUC of 0.67^37^. Additionally, a recent meta-analysis showed that the commonly used cut-off for PCT of 0.5 ng/mL only had a sensitivity of 44% to identify patients with pneumonia who have a high risk of death^38^. The meta-analysis performed by Su *et al*. found that the pooled sensitivity and specificity of sTREM-1 for the prediction of mortality in infection were 0.75 (95% CI, 0.61–0.86) and 0.66 (95% CI, 0.54–0.75), respectively^39^. Elevated suPAR levels at the initial stage were significantly associated with a high risk of death, with a pooled RR of 3.37 (95% CI, 2.60–4.38). The pooled sensitivity and specificity of suPAR for mortality prediction in adult patients with bacteremia and sepsis were 0.70 and 0.72, respectively, which were similar to those of sTREM-1. The largest review, which included cohort studies enrolling a total of 1914 patients, suggested that the Acute Physiology and Chronic Health Evaluation II (APACHE II) score combined with the serum suPAR level could be a novel rule for predicting the outcome of in sepsis^26^. Therefore, the combination of suPAR and other biological markers or risk scores can improve the prediction of mortality for infections.

Compared with meta-analyses including randomized controlled trials, those including diagnostic studies may have more publication bias^40^. We detected publication bias in the prognostic studies reviewed here. The exclusion of conference abstracts and studies without enough data could contribute to the publication and reporting bias. Thus, the value of suPAR for prognosis might be overestimated. We observed a significant degree of heterogeneity in sensitivity among the studies analyzed. A meta-regression analysis revealed that the clinical setting and QUADAS score substantially affected the sensitivity of suPAR for the diagnosis of bacterial infections. For the analysis of prognostic studies, the study design significantly contributed to the heterogeneities of both sensitivity and specificity in the meta-regression analysis. In addition, despite no significant threshold effect in both diagnostic and prognostic studies, the cut-off point of suPAR could also account for the heterogeneity. As shown in Table 1, high cut-off points (≥6.5 ng/ml for diagnostic studies; ≥10 ng/ml for prognostic studies) indicated a lower sensitivity and higher specificity of suPAR for diagnosing and predicting the mortality of bacterial infections.

Several limitations exist in the current meta-analysis. First, the serum suPAR level is associated not only with infection, but also with other diseases, such as malignant tumor, atherosclerosis and acute kidney injury/failure. Although the included studies mainly enrolled patients with sepsis and/or bacteremia, the baseline characteristics (disease severity, comorbidities, concurrent infections with pathogens except for bacteria, etc.) of the study populations varied, so some confounding factors might exist in the included studies. However, we could not specifically perform further analysis because of the limited data. Second, the sample sizes of some subgroup analyses were relatively small, which increased the rate of type II error. Third, since we did not have access to the original data of each study, we could not determine the optimal cut-off point of suPAR for the diagnosis and prognosis of bacterial infection. Lastly, some degree of publication bias could reduce the prognostic power of suPAR.

In conclusion, our meta-analysis suggests that the serum suPAR level could be a potential marker of bacterial infection. However, its accuracy in discriminating sepsis from SIRS in critically ill adult patients was not better than that of PCT and sTREM-1. Additionally, the suPAR level at the initial stage of infection had a moderate diagnostic value for mortality prediction in patients with sepsis and bacteremia. A high suPAR level could indicate the need for more intense monitoring and treatment. However, considering the lack of a perfect unique biomarker, further investigation is required to evaluate the usefulness of suPAR combined with other biomarkers for improving diagnostic efficacy.

## Additional Information

**How to cite this article**: Ni, W. *et al*. Serum soluble urokinase-type plasminogen activator receptor as a biological marker of bacterial infection in adults: a systematic review and meta-analysis. *Sci. Rep.* **6**, 39481; doi: 10.1038/srep39481 (2016).

**Publisher's note:** Springer Nature remains neutral with regard to jurisdictional claims in published maps and institutional affiliations.

## Supplementary Material

Supplementary Information

## Figures and Tables

**Table 1:** Subgroup analysis of the diagnostic and prognostic value of suPAR based on different variables.

Study type	Variables	Studies, No. (Patients, No.)	Sensitivity (95%CI)	Specificity (95%CI)	+LR (95%CI)	−LR (95%CI)	DOR (95%CI)	AUC (95%CI)
Diagnostic value	Overall	9 (1237)	0.73 (0.58–0.84)	0.79 (0.73–0.83)	3.4 (2.4–4.7)	0.34 (0.21–0.57)	10 (5–22)	0.82 (0.79–0.85)
	Bacteremia	3 (416)	0.60 (0.53–0.67)	0.79 (0.73–0.84)	3.2 (2.4–4.2)	0.48 (0.37–0.63)	7 (4–18)	0.82 (0.71–0.92)
	Sepsis	7 (1062)	0.67 (0.53–0.79)	0.80 (0.72–0.86)	3.4 (2.1–5.3)	0.41 (0.26–0.65)	8 (3–20)	0.82 (0.78–0.85)
	Sepsis from SIRS	4 (481)	0.61 (0.53–0.68)	0.82 (0.63–0.93)	3.4 (1.4–8.5)	0.48 (0.34–0.67)	7 (2–25)	0.68 (0.64–0.72)
	Cut–off <6.5 ng/ml	4 (612)	0.82 (0.49–0.95)	0.74 (0.67–0.81)	3.2 (2.0–5.2)	0.25 (0.07–0.92)	13(2–76)	0.78 (0.74–0.81)
	Cut-off ≥6.5 ng/ml	5 (625)	0.65 (0.56–0.72)	0.82 (0.74–0.88)	3.6 (2.3–5.5)	0.43 (0.34–0.55)	8 (4–15)	0.77 (0.73–0.80)
Prognostic value	Overall	10 (3041)	0.70 (0.60–0.78)	0.72 (0.62–0.80)	2.5 (1.9–3.3)	0.42 (0.33–0.55)	6 (4–9)	0.77 (0.73–0.80)
	Bacteremia	3 (332)	0.64 (0.51–0.76)	0.83 (0.78–0.87)	3.5 (2.2–5.7)	0.39 (0.16–0.92)	11 (5–22)	0.84 (0.76–0.92)
	Sepsis	7 (2709)	0.71 (0.62–0.79)	0.66 (0.58–0.74)	2.1 (1.7–2.6)	0.44 (0.33–0.58)	5 (3–8)	0.74 (0.70–0.78)
	Cut-off <10 ng/ml	4 (498)	0.75 (0.65–0.83)	0.61 (0.50–0.71)	1.9 (1.4–2.6)	0.40 (0.27–0.61)	5 (2–10)	0.75 (0.71–0.79)
	Cut-off ≥10 ng/ml	6 (2543)	0.65 (0.50–0.78)	0.78 (0.66–0.86)	2.9 (2.0–4.2)	0.45 (0.31–0.63)	7 (4–11)	0.78 (0.74–0.81)

Abbreviations: CI, confidence interval; +LR, positive likelihood ratio; −LR, negative likelihood ratio; DOR, diagnositic odds ratio; AUC, area under the receiver operating characteristic curve.

## References

[b1] Donadello K., Scolletta S., Covajes C. & Vincent J. L. suPAR as a prognostic biomarker in sepsis. BMC Med. 10, 2 (2012). 22221662 10.1186/1741-7015-10-2 PMC3275545

[b2] Thuno M., Macho B. & Eugen-Olsen J. suPAR: the molecular crystal ball. Dis Markers. 27, 157–172 (2009). 19893210 10.3233/DMA-2009-0657 PMC3835059

[b3] Sier C. F. . Presence of urokinase-type plasminogen activator receptor in urine of cancer patients and its possible clinical relevance. Lab Invest. 79, 717–722 (1999). 10378514

[b4] Sandquist M. & Wong H. R. Biomarkers of sepsis and their potential value in diagnosis, prognosis and treatment. Expert Rev Clin Immunol. 10, 1349–1356 (2014). 25142036 10.1586/1744666X.2014.949675 PMC4654927

[b5] Toldi G. . Soluble urokinase plasminogen activator receptor (suPAR) in the assessment of inflammatory activity of rheumatoid arthritis patients in remission. Clin Chem Lab Med. 51, 327–332 (2013). 22718576 10.1515/cclm-2012-0221

[b6] Andersen E. S. . Twelve potential fibrosis markers to differentiate mild liver fibrosis from cirrhosis in patients infected with chronic hepatitis C genotype 1. Eur J Clin Microbiol Infect Dis. 30, 761–766 (2011). 21229279 10.1007/s10096-010-1149-y

[b7] Andersen O., Eugen-Olsen J., Kofoed K., Iversen J. & Haugaard S. B. suPAR associates to glucose metabolic aberration during glucose stimulation in HIV-infected patients on HAART. J Infect. 57, 55–63 (2008). 18328568 10.1016/j.jinf.2008.01.014

[b8] Perch M. . Serum levels of soluble urokinase plasminogen activator receptor is associated with parasitemia in children with acute Plasmodium falciparum malaria infection. Parasite Immunol. 26, 207–211 (2004). 15491469 10.1111/j.0141-9838.2004.00695.x

[b9] Henriquez-Camacho C. & Losa J. Biomarkers for sepsis. Biomed Res Int. 2014, 547818 (2014). 24800240 10.1155/2014/547818 PMC3985161

[b10] Backes Y. . Usefulness of suPAR as a biological marker in patients with systemic inflammation or infection: a systematic review. Intensive Care Med. 38, 1418–1428 (2012). 22706919 10.1007/s00134-012-2613-1 PMC3423568

[b11] Bone R. C. . Definitions for sepsis and organ failure and guidelines for the use of innovative therapies in sepsis. The ACCP/SCCM Consensus Conference Committee. American College of Chest Physicians/Society of Critical Care Medicine. Chest. 101, 1644–1655 (1992). 1303622 10.1378/chest.101.6.1644

[b12] Whiting P., Rutjes A. W., Reitsma J. B., Bossuyt P. M. & Kleijnen J. Thedevelopment of QUADAS: a tool for the quality assessment ofstudies of diagnostic accuracy included in systematic reviews. BMC Med Res Methodol. 3, 25 (2003). 14606960 10.1186/1471-2288-3-25 PMC305345

[b13] Kofoed K. . Use of plasma C-reactive protein, procalcitonin, neutrophils, macrophage migration inhibitory factor, soluble urokinase-type plasminogen activator receptor, and soluble triggering receptor expressed on myeloid cells-1 in combination to diagnose infections: a prospective study. Crit Care. 11, R38 (2007). 17362525 10.1186/cc5723 PMC2206456

[b14] Koch A. . Circulating soluble urokinase plasminogen activator receptor is stably elevated during the first week of treatment in the intensive care unit and predicts mortality in critically ill patients. Crit Care. 15, R63 (2011). 21324198 10.1186/cc10037 PMC3221996

[b15] Yilmaz G., Köksal I., Karahan S. C. & Mentese A. The diagnostic and prognostic significance of soluble urokinase plasminogen activator receptor in systemic inflammatory response syndrome. Clin Biochem. 44, 1227–1230 (2011). 21816136 10.1016/j.clinbiochem.2011.07.006

[b16] Hoenigl M. . Diagnostic accuracy of soluble urokinase plasminogen activator receptor (suPAR) for prediction of bacteremia in patients with systemic inflammatory response syndrome. Clin Biochem. 46, 225–229 (2013). 23159293 10.1016/j.clinbiochem.2012.11.004

[b17] Kaya S. . The significance of serum urokinase plasminogen activation receptor (suPAR) in the diagnosis and follow-up of febrile neutropenic patients with hematologic malignancies. Int J Infect Dis. 17, e1056–9 (2013). 23742830 10.1016/j.ijid.2013.04.004

[b18] Loonen A. J. . Biomarkers and molecular analysis to improve bloodstream infection diagnostics in an emergency care unit. PLoS One. 9, e87315 (2014). 24475269 10.1371/journal.pone.0087315 PMC3903623

[b19] Reichsoellner M., Raggam R. B., Wagner J., Krause R. & Hoenigl M. Clinical evaluation of multiple inflammation biomarkers for diagnosis and prognosis for patients with systemic inflammatory response syndrome. J Clin Microbiol. 52, 4063–4066 (2014). 25187630 10.1128/JCM.01954-14 PMC4313254

[b20] Barati M., Shekarabi M., Chobkar S., Talebi-Taher M. & Farhadi N. Evaluation of diagnostic value of soluble urokinase-type plasminogen activator receptor in sepsis. Arch Clin Infect Dis. 10, e26346 (2015).

[b21] Zeng M. . Clinical value of soluble urokinase-type plasminogen activator receptor in the diagnosis, prognosis, and therapeutic guidance of sepsis. Am J Emerg Med. 34, 375–380 (2016). 26615223 10.1016/j.ajem.2015.11.004

[b22] Wittenhagen P. . The plasma level of soluble urokinase receptor is elevated in patients with Streptococcus pneumoniae bacteraemia and predicts mortality. Clin Microbiol Infect. 10, 409–415 (2004). 15113317 10.1111/j.1469-0691.2004.00850.x

[b23] Huttunen R. . Plasma level of soluble urokinase-type plasminogen activator receptor as a predictor of disease severity and case fatality in patients with bacteraemia: a prospective cohort study. J Intern Med. 270, 32–40 (2011). 21332843 10.1111/j.1365-2796.2011.02363.x

[b24] Mölkänen T., Ruotsalainen E., Thorball C. W. & Järvinen A. Elevated soluble urokinase plasminogen activator receptor (suPAR) predicts mortality in Staphylococcus aureus bacteremia. Eur J Clin Microbiol Infect Dis. 30, 1417–1424 (2011). 21479972 10.1007/s10096-011-1236-8

[b25] Savva A. . Soluble urokinase plasminogen activator receptor (suPAR) for assessment of disease severity in ventilator-associated pneumonia and sepsis. J Infect. 63, 344–350 (2011). 21839112 10.1016/j.jinf.2011.07.016

[b26] Giamarellos-Bourboulis E. J. . Risk assessment in sepsis: a new prognostication rule by APACHE II score and serum soluble urokinase plasminogen activator receptor. Crit Care. 16, R149 (2012). 22873681 10.1186/cc11463 PMC3580738

[b27] Suberviola B., Castellanos-Ortega A., Ruiz Ruiz A., Lopez-Hoyos M. & Santibañez M. Hospital mortality prognostication in sepsis using the new biomarkers suPAR and proADM in a single determination on ICU admission. Intensive Care Med. 39, 1945–1952 (2013). 23949703 10.1007/s00134-013-3056-z

[b28] Donadello K. . Soluble urokinase-type plasminogen activator receptor as a prognostic biomarker in critically ill patients. J Crit Care. 29, 144–149 (2014). 24120089 10.1016/j.jcrc.2013.08.005

[b29] Tsirigotis P. . Thrombocytopenia in critically ill patients with severe sepsis/septic shock: Prognostic value and association with a distinct serum cytokine profile. J Crit Care. 32, 9–15 (2016). 26726794 10.1016/j.jcrc.2015.11.010

[b30] Alberti C. . Influence of systemic inflammatory response syndrome and sepsis on outcome of critically ill infected patients. Am J Respir Crit Care Med. 168, 77–84 (2003). 12702548 10.1164/rccm.200208-785OC

[b31] Jiyong J., Tiancha H., Wei C. & Huahao S. Diagnostic value of the soluble triggering receptor expressed on myeloid cells-1 in bacterial infection: a meta-analysis. Intensive Care Med. 35, 587–595 (2009). 18936908 10.1007/s00134-008-1333-z

[b32] Czura C. J. “Merinoff symposium 2010: sepsis”-speaking with one voice. Mol Med. 17, 2–3 (2011). 21246163 10.2119/molmed.2010.00001.commentary PMC3022986

[b33] Yu C. W. . Role of procalcitonin in the diagnosis of infective endocarditis: a meta-analysis. Am J Emerg Med. 31, 935–941 (2013). 23601504 10.1016/j.ajem.2013.03.008

[b34] Tang B. M., Eslick G. D., Craig J. C. & McLean A. S. Accuracy of procalcitonin for sepsis diagnosis in critically ill patients: systematic review and meta-analysis. Lancet Infect Dis. 7, 210–217 (2007). 17317602 10.1016/S1473-3099(07)70052-X

[b35] Wu Y. . Accuracy of plasma sTREM-1 for sepsis diagnosis in systemic inflammatory patients: a systematic review and meta-analysis. Crit Care. 16, R229 (2012). 23194114 10.1186/cc11884 PMC3672614

[b36] Zhang X., Liu D., Liu Y. N., Wang R. & Xie L. X. The accuracy of presepsin (sCD14-ST) for the diagnosis of sepsis in adults: a meta-analysis. Crit Care. 19, 323 (2015). 26357898 10.1186/s13054-015-1032-4 PMC4566362

[b37] Peschanski N. . Prognostic value of PCT in septic emergency patients. Ann Intensive Care. 6, 47 (2016). 27207179 10.1186/s13613-016-0146-4 PMC4875576

[b38] Liu D., Su L. X., Guan W., Xiao K. & Xie L. X. Prognostic value of procalcitonin in pneumonia: A systematic review and meta-analysis. Respirology. 2, 280–288 (2016). 10.1111/resp.12704 PMC4738441 26662169

[b39] Su L., Liu D., Chai W., Liu D. & Long Y. Role of sTREM-1 in predicting mortality of infection: a systematic review and meta-analysis. BMJ Open. 6, e010314 (2016). 10.1136/bmjopen-2015-010314 PMC4874109 27178971

[b40] Irwig L., Macaskill P., Glasziou P. & Fahey M. Meta-analytic methods for diagnostic test accuracy. J Clin Epidemiol. 48, 119–130; discussion 131–132 (1995). 7853038 10.1016/0895-4356(94)00099-c

