# Supplementary material for: Serum soluble urokinase-type plasminogen activator receptor as a biological marker of bacterial infection in adults: a systematic review and meta-analysis
Source: Sci Rep. 2016 Dec 19;6:39481. doi: 10.1038/srep39481 (PMC5172370; doi:10.1038/srep39481)
Supplement: Supplementary Information [file srep39481-s1.pdf]

# Serum soluble urokinase-type plasminogen activator receptor as a biological marker of bacterial infection in adults: a systematic review and meta-analysis

**Running title: Usefulness of suPAR for bacterial infection in adults**

Wentao Ni[1¶], Yuliang Han[2¶], Jin Zhao[1], Junchang Cui[1], Kai Wang[2*], Rui Wang[3], Youning Liu[1*]

[1] Department of Respiratory Diseases, Chinese PLA General Hospital, Beijing 100853, China.

[2] Department of Neurology, Chinese PLA 305 Hospital, Beijing 100017, China.

[3] Department of Clinical Pharmacology, Chinese PLA General Hospital, Beijing, 100853, China.

**Table S1. Characteristics of included studies**

| Study type | Author | Year | Country | Study design | Clinical setting | Type of infection | Baseline information (percent of patients) | Sample size | Mean or median age (years) | Prevalence (infection rate/mortality) | Tested sample | Measured time | Type of assay kit | Cut-off (ng/ml) | TP | FP | FN | TN | QUADAS scores |
|---|---|---|---|---|---|---|---|---|---|---|---|---|---|---|---|---|---|---|---|
| Diagnostic value of suPAR | Kofoed [13] | 2007 | Denmark | PR | ED, et al | SIRS with infection | Solid tumours and haematological malignancies (9.3%); HIV infection (11.3%) | 151 | 56 (20 to 94) | 63.6% | Plasma | D0 | Luminex | 2.7 | 34 | 18 | 62 | 37 | 13 |
| | Koch [14] | 2011 | Germany | PR | ICU | Sepsis | Diabetes mellitus (32.7%); liver cirrhosis (7.0%) | 273 | 64 (18 to 90) | 72.2% | Serum | D0 | ViroGates | 5.5 | 148 | 21 | 49 | 55 | 12 |
| | Yilmaz [15] | 2011 | Turkey | PR | NA | SIRS with infection | Trauma (14.1%); Diabetes mellitus (9.4%); malignancies (5.9%) | 138 | 43.6 | 61.6% | Plasma | D0 | ViroGates | 2.8 | 78 | 8 | 7 | 45 | 9 |
| | Hoenigl [16] | 2013 | Austria | PR | ED | SIRS with bacteremia | Malignancies (20.5%); Cardiovascular disease (25.8%) | 132 | 67.3 | 41.7% | Serum | D0 | ViroGates | 7.9 | 34 | 18 | 21 | 59 | 11 |
| | Kaya [17] | 2013 | Turkey | PR | NA | Neutropenic patients with infection | Hematologic malignancies (100%) | 50 | 46.8 | 45.0% | Serum | D1 | ViroGates | 5.87 | 18 | 10 | 0 | 22 | 9 |
| | Loonen [18] | 2014 | Netherlands | RR | ED | Bacteremia | NA | 125 | 62.2 | 16.0% | Serum | NA | ViroGates | 7.5 | 16 | 24 | 4 | 81 | 11 |

| | | | | | | | | | | | | | | | | | | | |
|---|---|---|---|---|---|---|---|---|---|---|---|---|---|---|---|---|---|---|---|
| | Reichsoellner [19] | 2014 | Austria | PR | ED | SIRS with positive blood culture | Impaired renal function (52.8%); malignancies (22.0%) | 159 | 65.9 | 69.2% | Plasma | D0 | ViroGates | 7.6 | 61 | 7 | 49 | 42 | 12 |
| | Barati [20] | 2015 | Iran | PR | ICU | Sepsis | Malignancies (12.0%); trauma (9.6%); renal diseases (2.4%) | 83 | 62.4 | 48.2% | Plasma | D1 | USCN Life Science | 8.45 | 26 | 11 | 14 | 32 | 12 |
| | Zeng [21] | 2016 | China | PR | ICU | Sepsis | NA | 126 | 59 | 73.9% | Plasma | D1 | USCN Life Science | 9.52 | 59 | 2 | 23 | 42 | 11 |
| Prognostic value of suPAR | Wittenhagen [22] | 2004 | Denmark | MPR | NA | Bacteraemia | NA | 141 | 64 | 17.0% (In-hospital) | Plasma | D1 | NA | 10 | 9 | 6 | 15 | 111 | 12 |
| | Huttunen [23] | 2011 | Finland | PR | NA | Bacteraemia | Diabetes mellitus (25%); malignancies (17%); Cardiovascular disease (31%) | 132 | 62 (18 to 93) | 13.6% (month) | Plasma | D1-4 | ViroGates | 11 | 15 | 27 | 3 | 87 | 12 |
| | Koch [14] | 2011 | Germany | PR | ICU | Sepsis | Diabetes mellitus (32.7%); liver cirrhosis (7.0%) | 197 | 65 (20 to 90) | 35.9% (ICU) | Serum | D0 | ViroGates | 6 | 38 | 55 | 22 | 82 | 12 |
| | Molkanen [24] | 2011 | Finland | PR | NA | Bacteremia | Diabetes mellitus (24%); malignancies (19%); Chronic renal failure (26); Cardiovascular | 59 | NA | 32.2% (month) | Serum | D2-5 | ViroGates | 9.25 | 15 | 13 | 4 | 27 | 11 |

| | | | | | | | | | | | | | | | | | | |
|---|---|---|---|---|---|---|---|---|---|---|---|---|---|---|---|---|---|---|
| | | | | | | disease (36%) | | | | | | | | | | | | |
| Savva [25] | 2011 | Greece | MPR | ICU, et al | VAP with sepsis | Diabetes mellitus (15%); Cardiovascular disease (21%) | 180 | 58.4 | 27.8% (month) | Serum | D1 | ViroGates | 12.9 | 29 | 26 | 22 | 103 | 11 |
| Giamarellos [26] | 2012 | Greece | MPR | ICU, et al | Sepsis | Diabetes mellitus (22.5%); Cardiovascular disease (16.4%); chronic renal disease (9.2%) | 1914 | 66.1 | 21.9% (month) | Serum | D1 | ViroGates | 12 | 261 | 467 | 158 | 1028 | 12 |
| Subervi ola [27] | 2013 | Spain | PR | ICU | Severe sepsis and septic shock | ARDS (40.9%); CVVHDF (18.2%) | 137 | 62.6 | 29.9% (In-hospital) | Serum | D0 | ViroGates | 9.7 | 33 | 52 | 8 | 44 | 9 |
| Donadello [28] | 2014 | Belgium | PR | ICU | Sepsis | NA | 94 | 62 ±9 | 18.1% (ICU) | Serum | D0 | ViroGates | 10.2 | 12 | 27 | 5 | 50 | 9 |
| Tsirigotis [29] | 2016 | Greece | PR | ICU | Severe sepsis and septic shock | Diabetes mellitus (22.9%); malignancies (16.2%); Chronic renal failure (12.4%) | 105 | NA | 36.2% (month) | Serum | D1 | ViroGates | 7.6 | 31 | 18 | 7 | 49 | 10 |
| Zeng [21] | 2016 | China | PR | ICU | Sepsis | Diabetes mellitus; Cardiovascular disease | 82 | 58.74 ± 1.91 | 37.8% (month) | Plasma | D1 | USCN Life Science | 12.01 | 27 | 14 | 4 | 37 | 11 |

Abbreviations: PR, prospective recruitment; RR, retrospective recruitment; MPR, multicentre prospective recruitment; ED, emergency department; ICU, intensive care units; SIRS, Systemic Inflammatory Response Syndrome; ARDS, Acute Respiratory Distress Syndrome; CVVHDF, continuous veno-venous hemodialysis filtration; NA, not available; TP, true positive; FP, false positive; TN, true negative; FN, false negative.

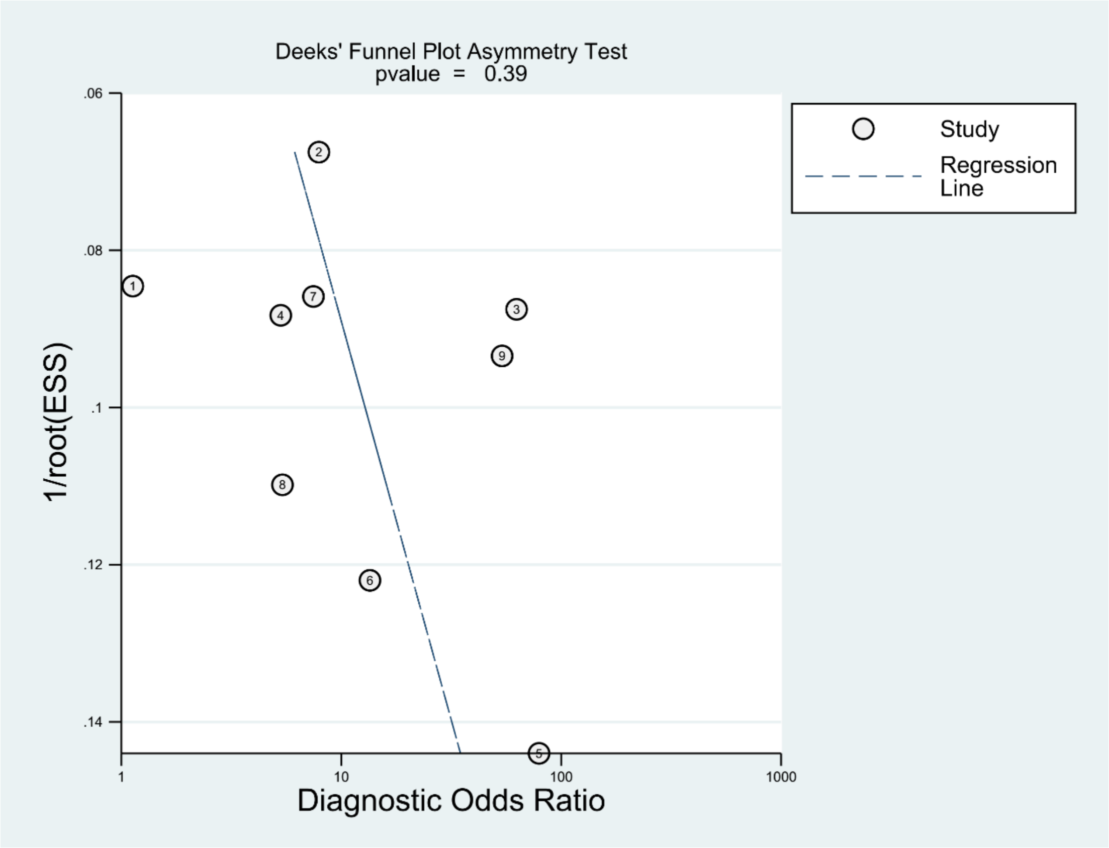


Figure S1. Deek's funnel plot asymmetry test for publication bias of studies evaluating the value of suPAR for the diagnosis of bacterial infections.

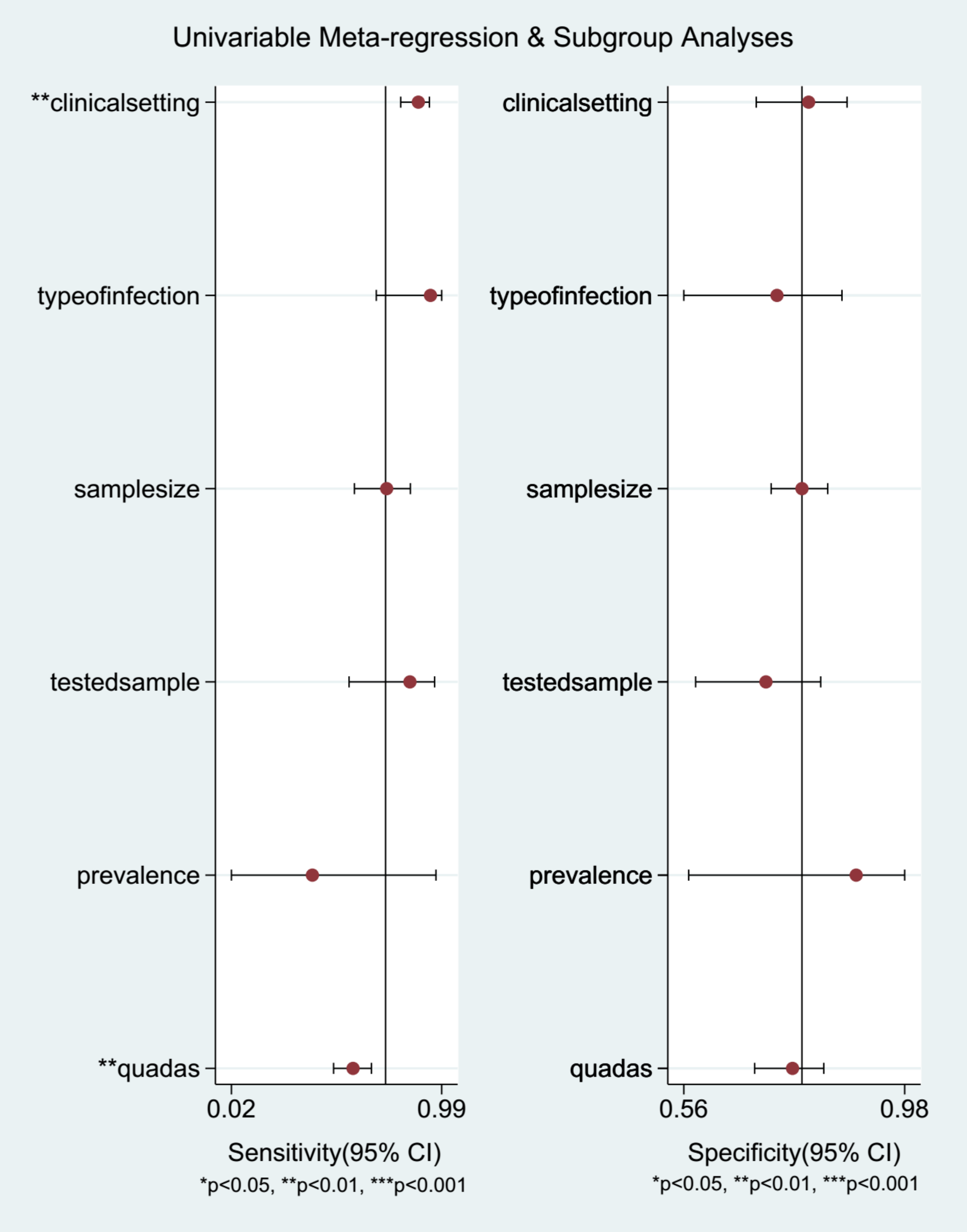


Figure S2. Univariate meta-regression and subgroup analysis of studies evaluating the value of suPAR for the diagnosis of bacterial infections.

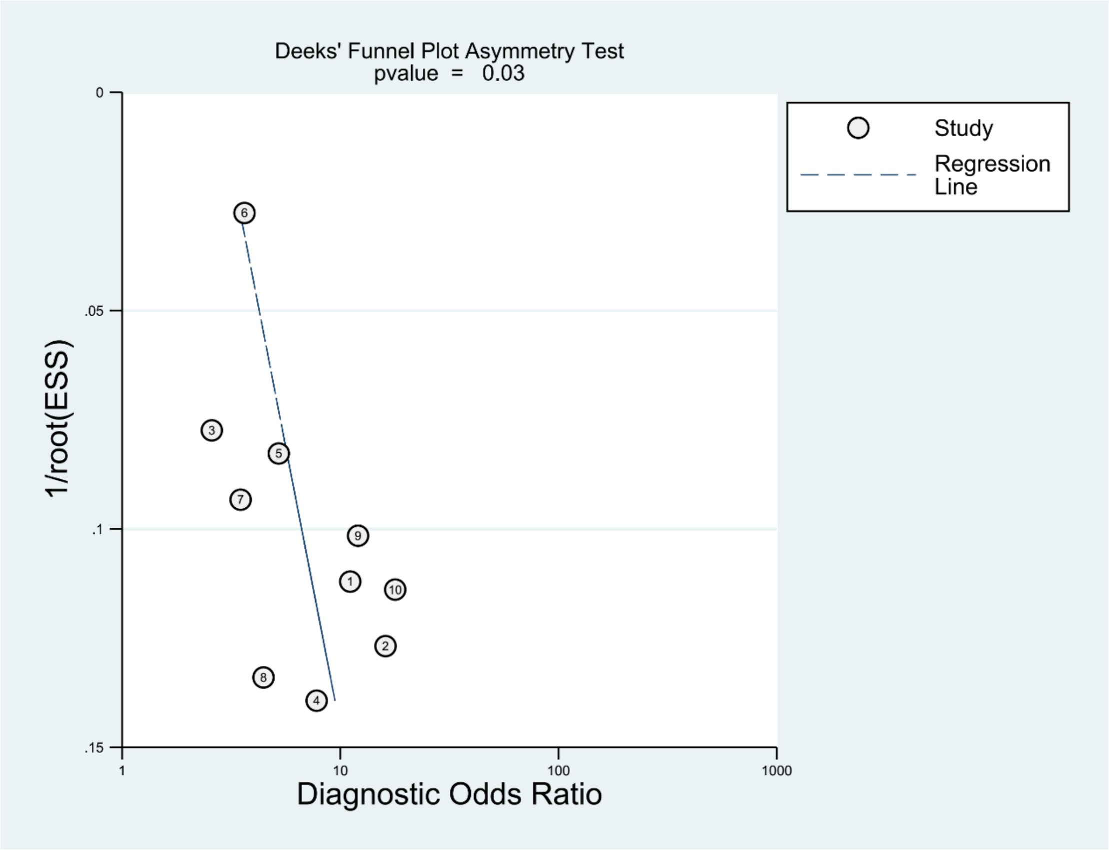


Figure S3. Deek's funnel plot asymmetry test for publication bias of studies evaluating the value of suPAR for the prediction of mortality in bacterial infections.

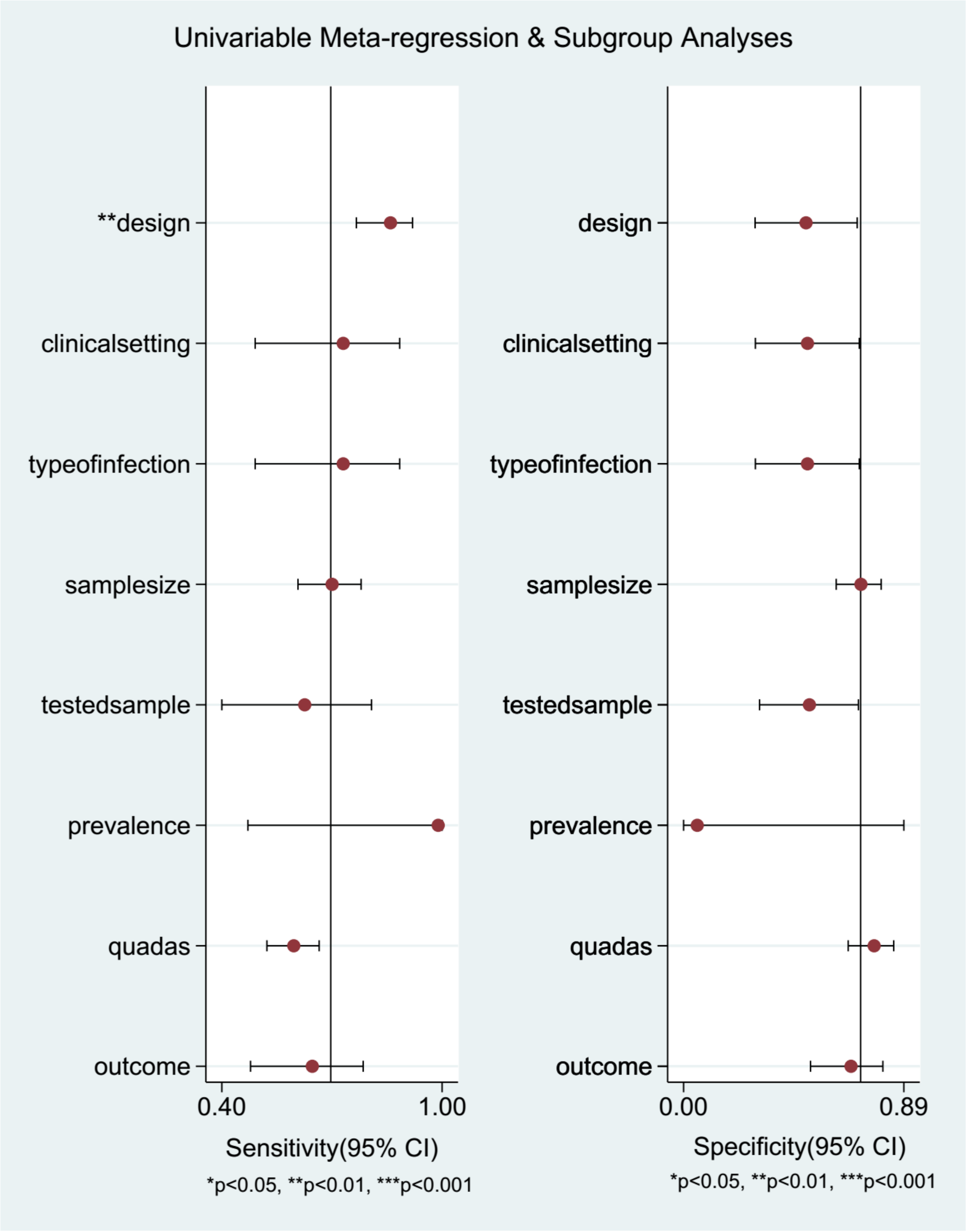


Figure S4. Univariate meta-regression and subgroup analysis of studies evaluating the value of suPAR for the prediction of mortality in bacterial infections.
